# Capabilities, opportunities and motivations for integrating evidence-based strategy for hypertension control into HIV clinics in Southwest Nigeria

**DOI:** 10.1371/journal.pone.0217703

**Published:** 2019-06-06

**Authors:** Juliet Iwelunmor, Oliver Ezechi, Chisom Obiezu-Umeh, Titilola Gbajabiamila, Adesola Z. Musa, David Oladele, Ifeoma Idigbe, Aigbe Ohihoin, Joyce Gyamfi, Angela Aifah, Babatunde Salako, Olugbenga Ogedegbe

**Affiliations:** 1 College for Public Health & Social Justice, Saint Louis University, Saint Louis, Missouri, United States of America; 2 Nigerian Institute of Medical Research, Yaba, Lagos, Nigeria; 3 New York University College of Global Public Health, New York, New York, United States of America; 4 Department of Population Health, New York University School of Medicine, New York, New York, United States of America; University of Ghana College of Health Sciences, GHANA

## Abstract

**Background:**

Given the growing burden of cardiovascular diseases in sub-Saharan Africa, global donors and governments are exploring strategies for integrating evidence-based cardiovascular diseases prevention into HIV clinics. We assessed the capabilities, motivations and opportunities that exist for HIV clinics to apply evidence-based strategies for hypertension control among people living with HIV (PLHIV) in Nigeria.

**Methods:**

We used a concurrent Quan-Qual- study approach (a quantitative first step using structured questionnaires followed by a qualitative approach using stakeholder meetings).We invited key stakeholders and representatives of HIV and non-communicable disease organizations in Lagos, Nigeria to 1) assess the capacity of HIV clinics (n = 29) to, and; 2) explore their attitudes and perceptions towards implementing evidence-based strategies for hypertension management in Lagos, Nigeria (n = 19)The quantitative data were analyzed using SPSS whereas responses from the stakeholders meeting were coded and analyzed using thematic approach and an implementation science framework, the COM-B (Capabilities, Opportunities, Motivations and Behavior) model, guided the mapping and interpretation of the data.

**Results:**

Out of the 29 HIV clinics that participated in the study, 28 **clinics were** public, government-owned facilities with 394 HIV patients per month with varying capabilities, opportunities and motivations for integrating evidence-based hypertension interventions within their services for PLHIV. Majority of the clinics (n = 26) rated medium-to-low on the psychological capability domains, while most of the clinics (n = 25) rated low on the physical capabilities of integrating evidence-based hypertension interventions within HIV clinics. There was high variability in the ratings for the opportunity domains, with physical opportunities rated high in only eight HIV clinics, two clinics with a medium rating and nineteen clinics with a low rating. Social opportunity domain tended to be rated low in majority of the HIV clinics (n = 21). Lastly, almost all the HIV clinics (n = 23) rated high on the reflective motivation domain although automatic motivations tended to be rated low across the HIV clinics.

**Conclusion:**

In this study, we found that with the exception of motivations, the relative capabilities whether physical or psychological and the relative opportunities for integrating evidence-based hypertension intervention within HIV clinics in Nigeria were minimal. Thus, there is need to strengthen the HIV clinics in Lagos for the implementation of evidence-based hypertension interventions within HIV clinics to improve patient outcomes and service delivery in Southwest Nigeria.

## Introduction

Despite decreases in overall new infections globally, HIV remains a major public health threat in many parts of sub-Saharan Africa (SSA).[1] For example, nearly half of all new HIV infections are in Nigeria, South Africa, and Uganda where HIV/AIDS is the highest cause of life-years lost.[2] The successful and efficient dissemination of highly active antiretroviral treatment (HAART) in SSA has contributed to the increased survival of PLHIV, with dramatic reductions in the AIDS-related morbidity and mortality.[3] Consequently, many countries in SSA are now faced with the double burden of HIV and non-communicable diseases (NCDs), such that the prevalence of hypertension has increased among PLHIV in SSA.[4] For example, the prevalence of hypertension in Nigeria among HIV-positive patients under anti-retroviral therapy ranges between 27%-41%.[5, 6] Thus, there is a strong need to reduce the burden of hypertension among PLHIV in Nigeria which requires the implementation of proven evidence-based interventions (EBIs) targeted at hypertension control in the region.

HIV clinics represent a key platform for implementing EBIs targeted at hypertension control in PLHIV. Nigeria has accomplished exemplary work focused on integrating management of NCDs (including screening and treatment of NCDs) into HIV care in HIV treatment centers. For example, several studies in Nigeria described the integration of cervical cancer screening into HIV clinicsfor its patient population.[7, 8] Of the 834 women offered cervical cancer screening at the Nigerian Institute of Medical Research (NIMR) HIV Treatment Center from 1st to 30th of April 2011, 96.5% accepted and 6.5% screened positive for cervical abnormalities.[8] This strategy can potentially be adopted for management of CVD risk factor like hypertension given its growing burden among PLWH in Nigeria.

Capacity, defined as the ability of a public health agency to provide or perform essential public health services, is a well-established factor necessary for the translation of EBIs into practice as well as their adoption and sustainability.[9, 10] There is little understanding of how HIV clinics in SSA countries operationalize, build, and maintain capacity, particularly as it relates to translation of EBIs into practice.[11] Because of the dynamic nature of the contexts of HIV care in SSA, an assessment of the capacity of HIV clinics to manage NCDs may provide stakeholders with the knowledge needed for a systematic planning of its implementation.

**Fig 1:**
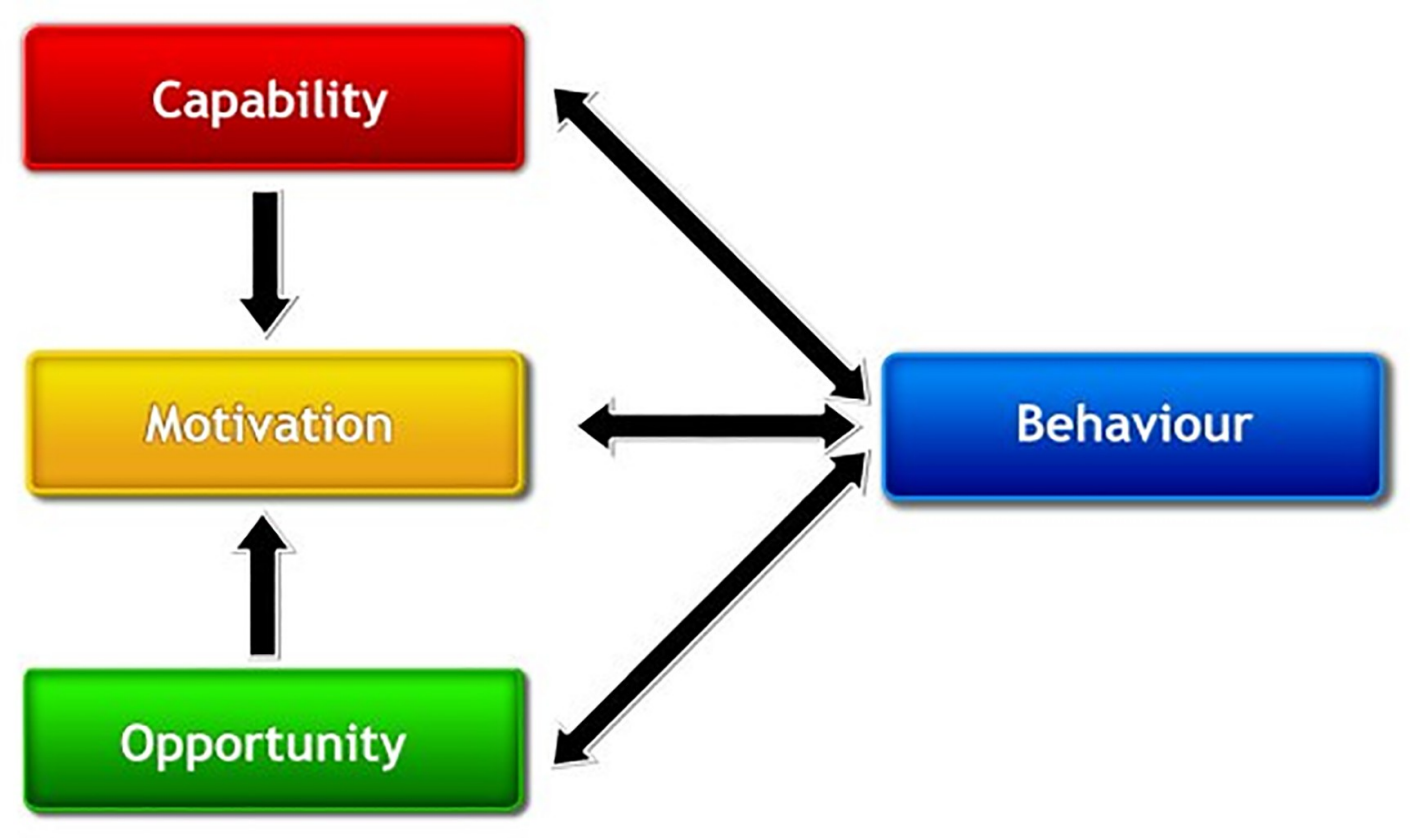
The COM-B model.

Given that HIV and hypertension are currently major public health issues in Nigeria, we adapted Mickie and colleagues Behavior Change Wheel framework [12], the COM-B model (Capabilities, Opportunities, Motivations and Behavior) [refer to Fig 1] to examine the capabilities, opportunities, and motivations for integrating evidence-based strategy for hypertension control within HIV clinics in Lagos, Nigeria. Although the COM-B model has been applied in other health contexts as a basis for developing effective interventions, it has not been applied to studies designed to integrate hypertension control strategies within HIV clinics in SSA. Findings from this study will be used to develop interventions targeted at the implementation of EBIs for hypertension control in HIV clinics as well as their sustainability.

## Methods

### Study design and setting

This study was conducted in Lagos, Nigeria between October 2017 and January 2018 and led by the Nigerian Institute for Medical Research (NIMR). The study design was a mixed-methods design using a concurrent Quan-Qual approach (a quantitative first step using structured questionnaires followed by a qualitative approach using stakeholder meetings). The study was approved by the Ethics Committee of the Nigerian Institute of Medical Research (NIMR).

For the quantitative aspect, we used a modified practice assessment survey to determine the practice capacity of 29 geographically distinct HIV clinics (who are all affiliated with NIMR) across 20 different Local Government Areas in Lagos State. For the qualitative aspect, we convened a stakeholder’s meeting of 18 representatives of HIV and non-communicable disease organizations from Lagos State Ministry of Health, Lagos State Primary Health Care Development Agency, Lagos State AIDS Control Agency, and the Nigerian Institute of Medical research. The goal of the stakeholder’s meeting was to brainstorm with key leadership personnel, the best strategies for integrating EBIs for HTN control within HIV clinics in Lagos State. The HIV Clinics were purposely selected based on the provision of comprehensive ART services at clinic site, patient load including private, public/government, faith-based organizations, and primary facility level. We recruited clinic representatives and stakeholders based on their impact and involvement in the management of NCDs and HIV. The stakeholder groups included implementation partners from NIMR, Lagos State Ministry of Health representatives, NCD district coordinators, health communicators and public health government officials. Participating HIV clinic representatives who were invited to complete the survey included four physicians, fourteen medical officers, four matrons, four nurses/midwives and three community health officers.

#### Practice assessment survey

The practice assessment data were collected with two instruments. First, we assessed the capacity of HIV clinics to manage NCDs by use of an adapted version of the Service Availability and Readiness Assessment (SARA) tool[13], which was developed by the World Health Organization and used in a similar study conducted in Tanzania[14]. Capacity of the HIV clinics and clinical management practices related to HTN care were assessed as follows: 1) Demographics (provider’s age, educational level, nature of facility, years of experience in HIV care), 2) Organizational characteristics (proportion of HIV patients with diagnosis of HTN, availability of tools for diagnosis and treatment of hypertension, financing mechanism), 3) Healthcare provider characteristics (case diagnosis, treatment, referral and clinic follow-up patterns), 4) Patient characteristics (access to information related to hypertension management and lifestyle behaviors). Secondly, the HIV clinic representatives completed a self-reported scale to assess the organizational context for implementing evidence-based interventions [15]—using a 14-item measure that assesses the degree to which evidence-based practices for hypertension management are implemented within the HIV clinics. The scale was further divided into 5 subscales: three were developed to focus primarily on evidence-practice for hypertension treatment, three to focus on educational support for evidence-based practice, three to focus on recognition for evidence-based practice, two of the items on reward and the last three items focus on openness to implementation. The questions were stated in English. The practice assessment tools used were further adapted based on feedback from pilot testing within a sub-set of participants at two HIV clinics (including NIMR). The survey items and statements were mapped to the domain of the implementation science framework COM-B (see Table 1).

**Table 1:** Mapping of survey questions and focus group discussion to the COM-B domains.

COM-B Domain	Questions/Statements
**Capability: The psychological and physical capacity of HIV clinics to integrate EBIs for hypertension control**	What is the first-line of anti-hypertensive drug choice for hypertensive treatment?
	Who is responsible ***for identifying*** hypertensive HIV patients in your facility?
	Who is responsible for ***the treatment*** of hypertensive HIV patients in your facility?
	Who is responsible for ***referring*** hypertensive HIV patients in your facility?
	Who is responsible for the ***follow-up care*** of hypertensive HIV patients in your facility?
	How are patient’s anthropometric measurements taken in your facility?
	How often are hypertensive patient’s blood pressures taken?
	What type of life-style related information does the facility provide to HIV patients for the management of hypertension?
**Opportunity: The environmental or contextual factors within the clinics that allow the use of proven hypertension interventions**	Which of the following investigative approach is used for the assessment of hypertensive HIV patients? (i.e. Urine Analysis, Blood glucose, Serum electrolytes, Total cholesterol, electrocardiogram)
	What anti-hypertensive drugs are available for the HIV patients at your facility?
	How often are anti-hypertensive drugs readily available at your facility?
	How do most HIV patients at your facility cover the cost of hypertension treatment or consultation?
	What type of Sphygmomanometers is used to check patient’s blood pressure?
	What are the sources of knowledge of the beneficial effects of living a healthy lifestyle among hypertensive HIV patients?
	The facility provides specific conferences, workshops or seminars on evidence-based practices for hypertension management and control.
	The facility provides evidence-based practice for hypertension treatment in-service training.
	Training materials, journals and other educational resources on evidence-based practices for hypertension management and control are provided.
**Motivation: Automatic or reflective mechanisms that enhance or inhibit the likelihood of utilizing evidence-based solutions for hypertension control within clinics**	Providers in this facility who utilize evidence-based practices for hypertension treatment are more qualified.
	One of the main goals of this facility is to effectively use evidence-based practices for hypertension management and control in HIV patients.
	Evidence-based practices for hypertension treatment are important to the providers in this facility.
	Using evidence-based for hypertension treatment is a top priority for the providers in this facility.
	Providers in this facility who utilize evidence-based practices for hypertension treatment are highly recommended.
	The providers in this facility who utilize evidence-based practices for hypertension treatment are well respected among their peers.
	Providers are financially incentivized to use evidence-based practices for hypertension treatment.
	The more a provider use evidence-based practices, the more likely they are to get a commission.
	This facility favors providers who are ***adaptable*** to the guidelines for hypertension treatment.
	This facility favors providers who are ***flexible*** to the guidelines for hypertension treatment.
	This facility favors providers who are open to new types of interventions for hypertension management and control.
	What are the responses of hypertensive HIV patients regarding the beneficial effects of the lifestyle-related information (i.e. heart-healthy diet, no tobacco products, physical activity, low sodium intake, reduce coffee intake)?

#### Key stakeholder meeting

In November 2017, we conducted a stakeholder meeting at NIMR with key stakeholders mentioned above. Participants were asked to describe the factors that will prompt the integration of HTN care into HIV clinics in Nigeria, as well as factors that will make it difficult to integrate HTN care within HIV clinics. They were asked to explain the reasoning for their suggestions, and to describe the best strategies to implement NCD care within the HIV program. The meeting, which lasted for two hours, was digitally recorded.

### Data analysis

The qualitative and qualitative data from the practice assessment survey and stakeholders meeting were analyzed independently. Responses to the practice assessment survey were entered in an excel spreadsheet and descriptive statistics were conducted using SPSS (Statistical Package for Social Sciences) software version 25. The data from the stakeholders meeting (in audio-recording device) were first transcribed and then analyzed using content analysis method. The emerging themes were analyzed using thematic analysis based approach on constructs of the COM-B model that was used to guide the interpretation of data. To ensure triangulation, two of the authors utilized the open and thematic coding process and continually worked back and forth between data sources. Discrepancies between the two coders were resolved by open discussions and consultation sessions among the research team.

**Fig 2:**
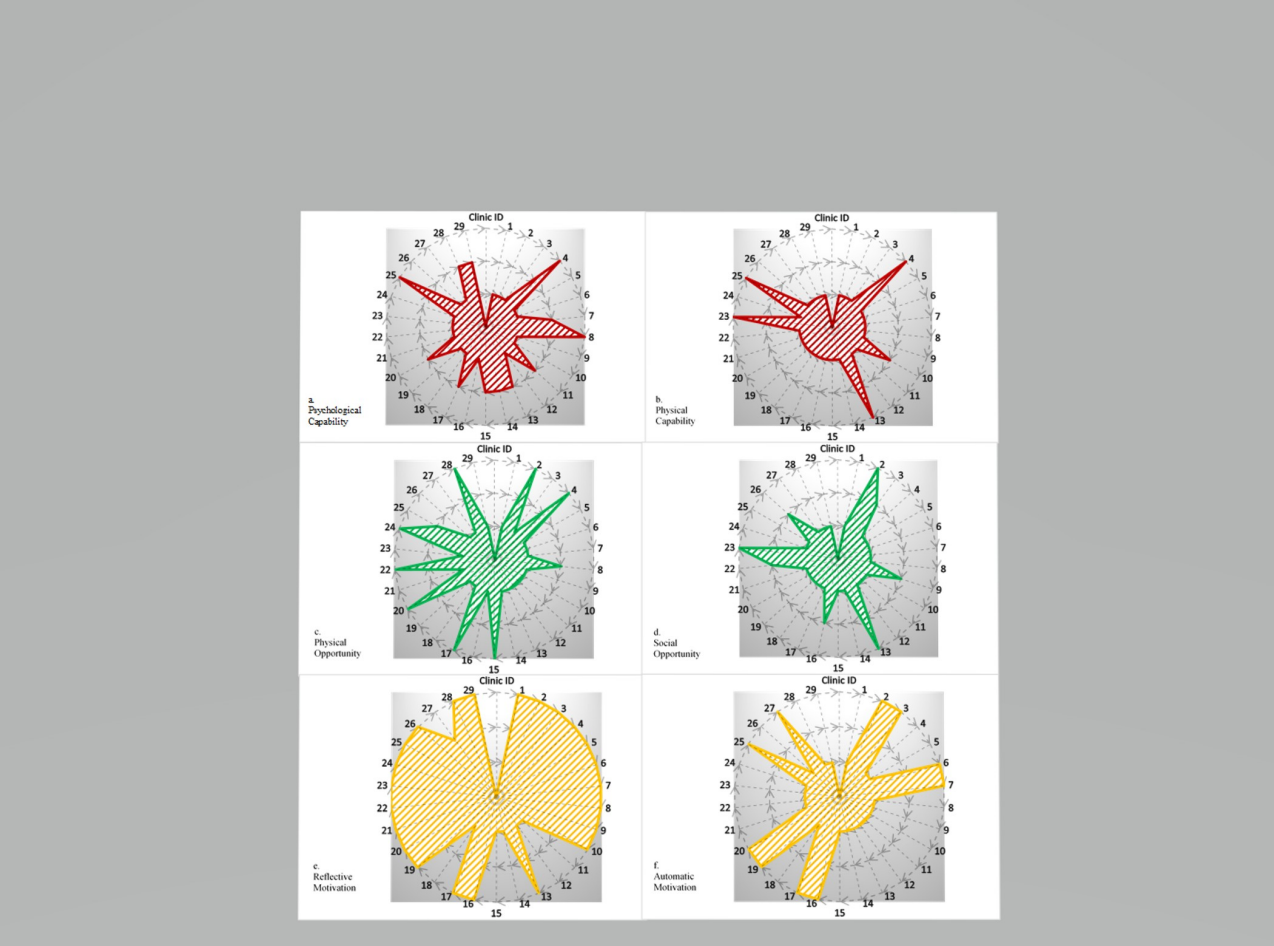
Capability, opportunity and motivation ratings of 29 geographically distinct HIV clinics in Lagos. Notes: (1) Responses to the practice assessment questionnaire were mapped against the COM-B components (capability, opportunity and motivation) (2) Each of the clinics were rated on a 3-point scale (low (the most inner radius), medium (middle radius) and high (outer radius)).

The responses to the practice assessment questionnaire were mapped against the COM-B components using radical charts to display the ratings of each of the HIV clinics, as outlined in Fig 2. For this mapping activity, we examined the two types of domains within each of the COM-B themes in order to have a more comprehensive and coherent schema to better formulate a “behavioral diagnosis” [16] towards integrating hypertension care within HIV clinics. A simple, 3-point rating score (low, medium, high) was used to gauge the strengths of each HIV clinic within each COM-B component. The smaller radius indicates low rating for the COM-B domain across all the HIV clinics, whereas the larger radius indicates high ratings.

## Results

### Study participants

The HIV clinics were mostly described as public, government-owned (97%) facilities. On average, the clinic providers care for an average of 394 HIV patients per month. Overall, 62% of the providers were female with a median age of 40 years. The median number of years that the providers have been in practice was six years. The estimated prevalence of hypertension among the HIV patients served at the local HIV clinics ranged from 0% to 31%. Out of the 18 participants who attended the stakeholders meeting, majority of the them were physicians (n = 12) within HIV clinics.

### Mapping of the survey responses for the HIV clinics to the COM-B model

Mapping of the HIV clinics to COM-B components yielded a wide-range of variability in terms of capabilities, opportunities and motivations for integrating evidence-based hypertension interventions into HIV clinics. Notably, the majority of the HIV clinics rated medium-to-low on the psychological capability domains (Fig 2A), with the exception of three clinics (Clinic ID #4, #8 and #25) which rated high on the psychological capability domain. Similarly, almost all the clinics rated low on the physical capability domain (Fig 2B) except four (Clinic ID #4, #13, #23 and #25) which rated high. There was high variability in the ratings for the opportunity domains. For the construct of physical opportunity, 8 clinics were rated high, two had a medium rating and 19 had a low rating (Fig 2C). For the social opportunity domain (Fig 2D), the majority of the clinics had a low rating. Almost all the clinics rated high on the reflective motivation domain although automatic motivations was rated low across the clinics (Fig 2E and 2F).

In addition to the mapping exercise, Table 2 summarizes how each of the themes generated from the qualitative stakeholder meeting, which was described in the context of barriers and enablers of integrating EBIs for hypertension control within HIV clinics, align with each of the COM-B domains.

**Table 2:** Mapping of COM-B components to the barriers and facilitators of integrating EBIs for hypertension management within HIV Clinics in Lagos, Nigeria.

COM-B domain		Theme
**Capability**	Barrier	Limited health education and training on NCD prevention and care Knowledge-to-skill gap Low self-efficacy and engagement
	Enabler	Task shifting of clinical duties
**Opportunity**	Barrier	Out of pocket payment Absence of evidence-based policies and guidelines Limited availability of basic diagnostic equipment and medication
	Enabler	Strengthening the referral network Compliancy with existing National Guidelines
**Motivation**	Barrier	Overburdened staff workload Lack of incentives for continuous adoption and enthusiasm
	Enabler	Perceived benefit of the intervention Supportive supervision Professional development

#### Capabilities for integrating hypertension management into HIV clinics

Low capability on health education and training on provision of NCD care. While all stakeholders agreed that basic education and trainings are key enabling factors to increasing knowledge of the healthcare workers, a few explained that there were inadequate or intermittent training opportunities for the healthcare workers at the HIV clinics: *“a lot of the clinics do not do refresher courses for their HIV practitioners…much less for hypertension care*.*”* Nonetheless, about half of the clinic representatives surveyed (41% to 52%), reported that the potential for educational support for evidence-based practice for hypertension treatment exists and should also be provided through specific conferences, workshops or seminars, in-service training, journals and other educational resources that are often available at their HIV clinics. This observation indicative of a knowledge-to-skill-gap within some of the clinics. One of the stakeholders presented a scenario where bridging the knowledge-to-skill gap may occur via training. For example, the stakeholder shared that the training administered to the nurses presented them the opportunity to enhance their skills beyond their expectations: *“We observed when we had an onsite training…it was an eye opener with the nurses saying “oh they didn't know that they could do this”*. *So maybe when we have training on these evidence-based hypertension interventions*, *it might be different*.*”* One stakeholder also shared that providing the training may positively influence the self-efficacy and engagement of the healthcare workers with the inclusion of hypertension prevention and control for people living with HIV: *“If we really train them well and show them the easy way to address the major issues people face…in hypertension care and also in HIV care…it will go a long way to improve their self-efficacy and how they engage with PLHIV coping with hypertension as well*.*”*

Need for task shifting of clinical duties. Given the limited availability of skilled nurses and doctors, the primary care clinics are unable to cope with the growing number of HIV patients, as described below: *“There are already human resources challenges that we are currently facing*, *now*, *we want to pull the same set of human resources [physicians] that we said is not adequate to provide this specialized hypertension services to the target group*.*”* As a result, the stakeholders underscored the need to implement task shifting or task sharing within the primary healthcare centers which entails delegating clinical duties to non-physician healthcare workers. Another stakeholder suggested that other cadres of healthcare workers like community health workers (CHW) and junior community health workers (JCHW) should be utilized within the clinics in order to reduce the workload. In addition, the CHWs and JCHWs also connect well with community members. Nevertheless, progress is currently being made in the implementation of task sharing within the healthcare system, as mentioned below: *“The new guideline (task-sharing) is trying to sensitize people to look at other health workers like CHW and the JCHW to come in to assist as we integrate these hypertension services because of work load for providing HIV care is already enormous*.*”*

#### Opportunities for integrating hypertension interventions within HIV clinics

Absence of evidence-based policies and guidelines on task-shifting of primary care duties. While “task shifting” or “task sharing” of clinical duties was highlighted by the majority of the stakeholders as a possible enabler for reducing the workload from the physicians, one of the stakeholders raised a concern that policies and guidelines have not been implemented at the clinics to regulate the shifted tasks to include workload, roles and responsibilities of the community health workers: *“We are throwing so much at this people for them to manage so many people…The issue we have now is that we don't have a policy that says we can only have a health worker take care of 50 people max*, *and beyond 50 people no more”*.

The lack of evidence-based guideline for task-shifting of primary care duties may result in staff overcrossing their boundaries due to role ambiguity, as described by a stakeholder: *“Let us know what we are task shifting and let them know their limitations; if you train them and if you’re to support let them know where they stand*, *but when we are not allowed to monitor whatever we are task shifting*, *there is a tendency for us to go out of the way*.*”*

Furthermore, a vast majority of the stakeholders denoted that although there is currently a draft national guideline for non-communicable disease management in Lagos state, it has not been implemented and is outdated. However, there is an existing guideline for HIV management.

High out-of-pocket payments as a barrier to management of HTN. A key barrier that emerged in the discussion was the high out-of-pocket payment that most patients are subjected to. Only 7% of the HIV clinic representatives surveyed indicated that the anti-hypertensive medications provided to patients at their clinic is free of charge. Eighty-six percent of the HIV clinic representatives reported that almost all HIV patients at their facility covers the cost of hypertension treatment or consultation fully out-of-pocket and none indicated that treatment was covered under health insurance.

In the light of this issue of out-of-pocket payment, one of the stakeholders revealed that there is progress currently being made to mitigate this barrier across Lagos State: *“the Health Insurance scheme would be commencing soon and it would be mandatory for everyone in Lagos state to be part of the Scheme*. *The scheme would have short term*, *midterm and long term plans for HIV care as well as the identification of indigent patients*.*”*

Limited availability of basic diagnostic equipment and medication. Based on responses from the practice assessment survey, basic diagnostic tools for NCDs, such as urinalysis, plasma creatinine, serum electrolytes, total cholesterol, serum lipoprotein, and echocardiogram, was reported to be present in 17%-79% of the HIV clinics. This suggests discordant beliefs regarding the availability of diagnostic tools at the clinics. One stakeholder revealed that although there are few clinics with adequate diagnostic tool, most the time, HIV clinics outsource or partner with better equipped primary healthcare centers across the local government areas to provide diagnostic testing. Another stakeholder reported that in Lagos State, there are *“371 primary healthcare centers*, *and only 57 of the centers*, *which are called flagship primary health centers are fully functional*, *well stocked with drugs and equipment”*.

Strengthening the referral network. In the context of task sharing, stakeholders raised a suggestion for strengthening the referral network. They identified the specific need for using the CHW to track patients because they provide home-based care. Furthermore, they expressed their beliefs that including CHWs within the referral network could potentially provide a comprehensive healthcare system that begins at home and extends to healthcare facilities: *“These people should be involved because they are going to take some of the burden off the nurse’s work that he/she is complaining about and they will provide home-based care*. *As a result*, *we are going to be looking at a comprehensive system which starts right from the home down to the health system… which will further strengthen the referral network when you use the community health care workers*.*”*

Compliance with existing national guidelines. Compliance with existing National Reporting guidelines was proposed by one of the stakeholders to ensure fidelity and sustainability of the intervention: *“a guide*, *a scheme of service*, *an operational manual does not necessarily reflect operationality…the report coming from such systems may not be reflective of the actual activity on the field*. *Thus*, *it is important for the system of reporting now to be structured along with the national guidelines*.*”*

#### Motivation for integrating hypertension interventions within HIV clinics

Overburdening staff workload. Based on the practice assessment survey, more than 70% of the HIV clinic representatives indicated that the medical officers alone are responsible for identifying, treating, referring and the continuity of care for the HIV patients who are hypertensive. Thus, in-line with the commonly held concern of the stakeholders that doctor and nurses at the HIV clinic are overburdened with tasks: *“We have a clinic where we might have 3 doctors running a shift and we have some nurses but not so much*, *and if you talk to all of them they will tell you that they are overwhelmed*.*”* In addition, a few of the stakeholders cited their fear of the possibility of further straining the already overburdened workforce as a result of integrating NCD care into the HIV clinic: *“so if someone has comorbid condition you’re now going to train the health workers to provide hypertension care in an HIV setting*, *this may overburden the health workers*.*”* Particularly, a stakeholder reported that although health workers in the HIV clinics are willing to integrate, their current workload burden might make it difficult for them to adopt the intervention: *“The health workers want to do it; the problem is that they are just overwhelmed*.*”*

Lack of incentives for sustainability and enthusiasm. Reflecting on previous HIV funded programs, stakeholders recognized that one barrier to program sustainability and enthusiasm of health workers was the lack of incentives. Incentives were often discussed in relation to the provision of workshops, on-the job training, career advancement opportunities and monetary values. One stakeholder explained that: *“The issue of incentives killed the HIV programs in Nigeria… because the money is not coming in the way it used to be in the past*, *most health care workers are not going into HIV services*.*”* Thus, the discontinuation of supporting funds for HIV service delivery forces the clinicians at the HIV centers to explore other non-HIV care services. In addition, all the HIV clinic representatives surveyed were in total disagreement with the statement that ‘*providers are financially incentivized to use evidence-based practices for hypertension treatment*.’

Professional development. To address the issues of incentives, professional development opportunities were often cited by the stakeholders as a recommendation to be included in the intervention package: *“They want a sense of personal development*. *That is if I leave this position today as a community health worker*, *will people still call me*? *…So if there is a sense of training such that they can also have refresher courses*, *that will be considered as an incentive*.*”* Another stakeholder stressed the importance of issuing certification upon training completion as an example of valuable non-monetary incentive: *“But another thing for incentive is the training*, *and more importantly the certificates that you give to them*.*”*

Supportive supervision. As highlighted by one of the stakeholders, adequate staff supervision and support is necessary to boost the *“confidence level”* of the community healthcare workers to successfully engage in the evidence-based practices: *“The only problem now we have is that people don't supervise people; for example*, *if we task shift and I am told to supervise*, *instead of me to support them*, *I will be looking for their mistakes to discredit their training; but if you do supportive supervision their confidence level will go up*!*”*

Perceived benefit of the integration. The stakeholders believed that integrating NCDs into HIV care will reduce stigmatization and wait time by ensuring patients can walk into any General Out-Patients Department (GOPD), where all the patients’ complaint can be addressed in one location, without patients knowing each other’s diagnosis. Although, “*not all the practices in the states have adopted integration of services right now”*, however, some of the sub-facilities are currently undergoing integration. They also mentioned that all doctors will have the ability to provide any service anywhere within the facility at the primary and secondary care level if services are integrated: *“We should look towards more of integration of HIV services into primary care… because aside from reducing stigma*, *it will reduce the waiting time…there would be a pool of Doctors in GOPD available to attend to the patients*.*”*

## Discussion

This study examined the capabilities, opportunities, and motivations for integrating evidence-based hypertension interventions for people living with HIV within HIV clinics in Southwest Nigeria. Using the COM-B model (Capabilities, Opportunities, and Motivations) as a guide, we identified how factors such as education and training to reduce the knowledge-to-skill gap and boost the level of self-efficacy, task sharing of clinical duties to reduce the overburdened staff workload, limited availability and use of evidence-based guidelines, access to basic diagnostic tools and medications, and the availability of professional development opportunities may influence the implementation, delivery, and expansion of integrated hypertension services for PLHIV within HIV clinics. Although previous studies conducted in sub-Saharan Africa (SSA) have made mention of similar barriers to integrating NCD management, majority of the studies focused on other chronic diseases (i.e. cervical cancer, mental health, diabetes) but not hypertension, which is characterized as a silent killer and is currently the leading risk factor for CVD.[17–21] The identification of barriers or enablers by themselves are also not a new undertaking [22]; however, our approach to the assessment of these barriers across multiple clinical sites is unique and may potentially inform the development of interventions targeting the capabilities, opportunities, and motivations for successful service delivery of evidence-based hypertension interventions within HIV clinics.

Overall, our findings suggest a broader evaluation of the implementation characteristics that could provide a greater understanding of which capabilities, opportunities, or motivations result in more rapid, and lasting implementation of evidence-based hypertension interventions within HIV clinics. Findings from mapping the practice assessment responses against the COM-B component on the radical charts was highly variable across the 29 HIV clinics, with few clinics having the capabilities, opportunities, and motivations to adequately tackle hypertension among people living with HIV, despite its prevalence, morbidity and relative ease of management. The low-to-medium variations in capabilities for example, may reflect the early phase of the HIV/NCD integration in Nigeria as well as in other countries in sub-Saharan Africa.[23–25] However, there is a need to explore, the implementation process, particularly the implementation outcomes necessary for example, for highlighting fidelity or whether the evidence-based hypertension interventions are implemented as designed and tested so that the success for non HIV populations can be replicated across patients living with HIV.

Other findings from the practice assessment survey and stakeholder meetings in relation to physical and social opportunities highlight the existing resource constraints and cultural barriers within the HIV clinics that may influence the integration of proven hypertension interventions. A major challenge to NCD management within the HIV clinics is the limited access to basic diagnostic equipment, availability of medication and evidence-based policies and guidelines, which is in-line with other studies conducted in SSA within primary care health facilities or HIV clinics.[14, 26–28] Some stakeholders noted that among all the 371 primary healthcare centers in Lagos state, only 15% (57/371 centers) of them are fully functional and are well stocked with drugs and equipments.[29, 30] Similar to study conducted in Nigeria, when medications are unavailable at the healthcare centers, patients are advised to purchase drugs from an outside source.[28] This problem maybe more pronounced in HIV clinics that are disjoined from the primary health centers because there is no guarantee that HIV patients will have the same level of access to cardiovascular diseases care and treatment when compared to other patients seen at the general hospitals. The survey showed that within some of these HIV clinics, basic lab testing such as urine analysis, plasma creatinine, serum electrolytes, total cholesterol, serum lipoprotein were readily available at the clinics.

In addition to the capabilities and opportunities, a key barrier to motivation identified is the lack of valuable incentives for continuous adoption and enthusiasm. For example, a stakeholder gave a scenario where discontinued supporting funds for HIV service delivery negatively impacted the healthcare workforce to the extent that clinicians were forced to explore other non-HIV care services. To solve the issue of incentives, stakeholders suggested the need for capacity building to foster staff morale and professional development opportunities within the HIV clinics that will enhance and advance the skills of the healthcare workers. In addition, supportive supervision was highlighted as an enabler to foster motivation and productivity at the HIV clinics. Iwelunmor and colleagues conducted a narrative synthesis of health interventions implemented in SSA and found that overtime, sustainability of interventions is likely to be affected where there is no proper supervision and supportive community environment.[31]

Major motivators, however, identified by stakeholders was their strong belief about the benefits of integrating NCDs such as evidence-based hypertension interventions into HIV care, which may help explain the reason behind the high ratings on the reflective function of the radical chart (see Fig 2E). Although a few initially expressed their concern of further overburdening the already fragile health system, majority agreed that integrating HTN care will not only reduce the double burden, but it will also increase access, enable healthcare workers to treat and cater to the healthcare needs of the HIV patients in one location at a single-entry point, which will potentially reduce stigmatization and wait time at the HIV clinics. This strategy is similar to the one-stop shop integrated healthcare delivery model which has been shown, in studies conducted in South Africa[32], Kenya[33], Nigeria[34] and Malawi[35], to improve HIV patient’s access to prevention and treatment resources.

We identified a number of strengths and limitations in our work. The primary strength was the use of the COM-B model to map the practice assessment surveys and stakeholder perspectives on the capabilities, opportunities and motivations for integrating evidence-based hypertension interventions within HIV clinics. Limitations of this study include limited generalizability across other HIV clinics within Nigeria’s 6 geopolitical regions, as this study was conducted in one geopolitical zone, the Southwest region of Nigeria. Nigeria is a diverse and complex nation, thus, our results may not uniformly apply to other geopolitical regions of the country. Additionally, the potential for respondent bias is a limitation as respondents may have provided desirable answers to the questions posed on integrating evidence-based hypertension interventions within HIV clinics. Although desirable answers could have been given in some instances, the use of a mapping assessment component using the COM-B radial charts, may limit the potential for this limitation. Finally, although a range of key healthcare personnel participated in the stakeholders meetings, it may have been beneficial to gauge the perspective of health workers from other cadres such as nurses, matrons, community health workers, pharmacists and volunteers involved with service delivery within HIV clinics. As a result, further research is needed to explore whether the barriers and facilitators identified by the key leadership representatives are similar to those identified by other cadres.

Despite these imitations, there are several strengths to this study. As mentioned earlier, we utilized several fundamental principles for implementation science research which included, the identification of research-to-practice gap, stakeholder and community engagement, partaking in formative research activities, and understanding the likely process for change using a comprehensive theoretical framework.[36] To our knowledge, this study is among the first in SSA to systematically identify key capabilities, opportunities, and motivations for integrating hypertension within HIV services in the context of the COM-B components. Our use of the COM-B model allowed our findings to be described in consistent implementation science terminology, which in turn may inform implementation of integrated hypertension control strategies across multiple contexts providing HIV services. Future research should evaluate how these components translate into the long-term success of strategies focused on integrating evidence-based hypertension interventions within HIV clinics in resource limited settings.

## Conclusion

Using the COM-B model, we have shown that while there are major modifiable capability and opportunity barriers to integrating hypertension control strategies within HIV clinics in Lagos, Nigeria, the motivation and willingness to integrate is apparent within the clinics. Taken together, the findings can inform decision makers on how to implement and ultimately scale-up and refine over time, integrated services for evidence-based hypertension interventions within HIV clinics.

## Supporting information

S1 Text Study questionnaire assessing the capabilities, opportunities and motivations for integrating evidence-based strategy for hypertension control into HIV clinics in Southwest Nigeria. (DOCX)

## Abbreviations

SSA: Sub-Saharan Africa
HTN: Hypertension
BP: Blood Pressure
HIV: Human Immunodeficiency Virus
AIDS: Acquired Immunodeficiency Syndrome
PLHIV: People living with HIV
CVD: Cardiovascular Disease
NIMR: Nigerian Institute of Medical Research
NCD: Non-communicable diseases
ART: Antiretroviral Treatment
CHEW: Community Healthcare Workers
JCHEW: Junior Community Healthcare Workers
COM-B: Capabilities, Opportunities, Motivations and Behavior
EBI: Evidence-Based Intervention

## References

[1] Kharsany AB, Karim QA. HIV Infection and AIDS in Sub-Saharan Africa: Current Status, Challenges and Opportunities. Open AIDS J. 2016;10:34–48. 10.2174/1874613601610010034 27347270 PMC4893541

[2] Haldane V, Legido-Quigley H, Chuah FLH, Sigfrid L, Murphy G, Ong SE, et al Integrating cardiovascular diseases, hypertension, and diabetes with HIV services: a systematic review. AIDS Care. 2018;30(1):103–15. 10.1080/09540121.2017.1344350 28679283

[3] Dimala CA, Atashili J, Mbuagbaw JC, Wilfred A, Monekosso GL. Prevalence of Hypertension in HIV/AIDS Patients on Highly Active Antiretroviral Therapy (HAART) Compared with HAART-Naive Patients at the Limbe Regional Hospital, Cameroon. PLoS One. 2016;11(2):e0148100 10.1371/journal.pone.0148100 26862763 PMC4749660

[4] Todowede OO, Sartorius B. Prevalence of metabolic syndrome, discrete or comorbid diabetes and hypertension in sub-Saharan Africa among people living with HIV versus HIV-negative populations: a systematic review and meta-analysis protocol. BMJ Open. 2017;7(7). 10.1136/bmjopen-2017-016602 PMC5726114 28694350

[5] Iwuala SO, Lesi OA, Olamoyegun MA, Sabir AA, Fasanmade OA. Lipoatrophy among patients on antiretroviral therapy in Lagos, Nigeria: Prevalence, pattern and association with cardiovascular risk factors. Niger J Clin Pract. 2015;18(5):626–32. 10.4103/1119-3077.154208 26096241

[6] Ekrikpo UE, Akpan EE, Ekott JU, Bello AK, Okpechi IG, Kengne AP. Prevalence and correlates of traditional risk factors for cardiovascular disease in a Nigerian ART-naive HIV population: a cross-sectional study. BMJ Open. 2018;8(7):e019664 10.1136/bmjopen-2017-019664 30030310 PMC6059292

[7] Odafe S, Torpey K, Khamofu H, Oladele E, Adedokun O, Chabikuli O, et al Integrating cervical cancer screening with HIV care in a district hospital in Abuja, Nigeria. Niger Med J. 2013;54(3):176–84. 10.4103/0300-1652.114590 23901180 PMC3719244

[8] Ezechi OC, Gab-Okafor CV, Ostergren PO, Odberg Pettersson K. Willingness and acceptability of cervical cancer screening among HIV positive Nigerian women. BMC Public Health. 2013;13(1):46. 23327453 10.1186/1471-2458-13-46 PMC3567931

[9] Leeman J, Birken SA, Powell BJ, Rohweder C, Shea CM. Beyond "implementation strategies": classifying the full range of strategies used in implementation science and practice. Implement Sci. 2017;12(1):125 10.1186/s13012-017-0657-x 29100551 PMC5670723

[10] Shelton RC, Cooper BR, Stirman SW. The Sustainability of Evidence-Based Interventions and Practices in Public Health and Health Care. Annu Rev Public Health. 2018;39(1):55–76. 29328872 10.1146/annurev-publhealth-040617-014731

[11] Ojo T, Lester L, Iwelunmor J, Gyamfi J, Obiezu-Umeh C, Onakomaiya D, et al Feasibility of integrated, multilevel care for cardiovascular diseases (CVD) and HIV in low- and middle-income countries (LMICs): A scoping review. PLoS One. 2019;14(2):e0212296 10.1371/journal.pone.0212296 30794591 PMC6386271

[12] Michie S, van Stralen MM, West R. The behaviour change wheel: a new method for characterising and designing behaviour change interventions. Implement Sci. 2011;6:42 10.1186/1748-5908-6-42 21513547 PMC3096582

[13] WHO. Service Availability and Readiness Assessment (SARA) [Available from: http://www.who.int/healthinfo/systems/sara_introduction/en/.

[14] Peck R, Mghamba J, Vanobberghen F, Kavishe B, Rugarabamu V, Smeeth L, et al Preparedness of Tanzanian health facilities for outpatient primary care of hypertension and diabetes: a cross-sectional survey. Lancet Glob Health. 2014;2(5):e285–92. 10.1016/S2214-109X(14)70033-6 24818084 PMC4013553

[15] Ehrhart MG, Aarons GA, Farahnak LR. Assessing the organizational context for EBP implementation: the development and validity testing of the Implementation Climate Scale (ICS). Implementation Science. 2014;9(1):157. 25338781 10.1186/s13012-014-0157-1 PMC4210525

[16] Atkins L, Michie S. Designing interventions to change eating behaviours. Proc Nutr Soc. 2015;74(2):164–70. 10.1017/S0029665115000075 25998679

[17] Matanje Mwagomba BL, Ameh S, Bongomin P, Juma PA, MacKenzie RK, Kyobutungi C, et al Opportunities and challenges for evidence-informed HIV-noncommunicable disease integrated care policies and programs: lessons from Malawi, South Africa, Swaziland and Kenya. AIDS. 2018;32 Suppl 1:S21–S32. 29952787 10.1097/QAD.0000000000001885

[18] Mall S, Sorsdahl K, Swartz L, Joska J. "I understand just a little …" Perspectives of HIV/AIDS service providers in South Africa of providing mental health care for people living with HIV/AIDS. AIDS Care. 2012;24(3):319–23. 10.1080/09540121.2011.608790 22273005

[19] Kumakech E, Andersson S, Wabinga H, Berggren V. Integration of HIV and cervical cancer screening perceptions of healthcare providers and policy makers in Uganda. BMC Public Health. 2014;14(1):810. 25099996 10.1186/1471-2458-14-810 PMC4246470

[20] Moon TD, Silva-Matos C, Cordoso A, Baptista AJ, Sidat M, Vermund SH. Implementation of cervical cancer screening using visual inspection with acetic acid in rural Mozambique: successes and challenges using HIV care and treatment programme investments in Zambezia Province. J Int AIDS Soc. 2012;15(2):17406 10.7448/IAS.15.2.17406 22713260 PMC3499800

[21] Rabkin M, Melaku Z, Bruce K, Reja A, Koler A, Tadesse Y, et al Strengthening Health Systems for Chronic Care: Leveraging HIV Programs to Support Diabetes Services in Ethiopia and Swaziland. J Trop Med. 2012;2012:137460 10.1155/2012/137460 23056058 PMC3465908

[22] Moore JE, Mascarenhas A, Marquez C, Almaawiy U, Chan WH, D'Souza J, et al Mapping barriers and intervention activities to behaviour change theory for Mobilization of Vulnerable Elders in Ontario (MOVE ON), a multi-site implementation intervention in acute care hospitals. Implement Sci. 2014;9:160 10.1186/s13012-014-0160-6 25928538 PMC4225038

[23] Kemp CG, Weiner BJ, Sherr KH, Kupfer LE, Cherutich PK, Wilson D, et al Implementation science for integration of HIV and non-communicable disease services in sub-Saharan Africa: a systematic review. AIDS. 2018;32 Suppl 1:S93–S105. 29952795 10.1097/QAD.0000000000001897

[24] Temu F, Leonhardt M, Carter J, Thiam S. Integration of non-communicable diseases in health care: tackling the double burden of disease in African settings. Pan Afr Med J. 2014;18:202 10.11604/pamj.2014.18.202.4086 25419329 PMC4237574

[25] Njuguna B, Vorkoper S, Patel P, Reid MJA, Vedanthan R, Pfaff C, et al Models of integration of HIV and noncommunicable disease care in sub-Saharan Africa: lessons learned and evidence gaps. AIDS. 2018;32 Suppl 1:S33–S42. 29952788 10.1097/QAD.0000000000001887 PMC6779053

[26] Leung C, Aris E, Mhalu A, Siril H, Christian B, Koda H, et al Preparedness of HIV care and treatment clinics for the management of concomitant non-communicable diseases: a cross-sectional survey. BMC Public Health. 2016;16(1):1002 10.1186/s12889-016-3661-1 27655406 PMC5031255

[27] Bintabara D, Mpondo BCT. Preparedness of lower-level health facilities and the associated factors for the outpatient primary care of hypertension: Evidence from Tanzanian national survey. PLoS One. 2018;13(2):e0192942 10.1371/journal.pone.0192942 29447231 PMC5814020

[28] Oyekale AS. Assessment of primary health care facilities' service readiness in Nigeria. BMC Health Serv Res. 2017;17(1):172 10.1186/s12913-017-2112-8 28249578 PMC5333428

[29] Aregbeshola BS, Khan SM. Primary Health Care in Nigeria: 24 Years after Olikoye Ransome-Kuti's Leadership. Front Public Health. 2017;5:48 10.3389/fpubh.2017.00048 28349050 PMC5346888

[30] UNICEF LSMoHa. Reducing Health Disparities in Lagos State: An Investment Case. 2012.

[31] Iwelunmor J, Blackstone S, Veira D, Nwaozuru U, Airhihenbuwa C, Munodawafa D, et al Toward the sustainability of health interventions implemented in sub-Saharan Africa: a systematic review and conceptual framework. Implementation Science. 2016;11(1):43. 27005280 10.1186/s13012-016-0392-8 PMC4804528

[32] Kerschberger B, Hilderbrand K, Boulle AM, Coetzee D, Goemaere E, De Azevedo V, et al The effect of complete integration of HIV and TB services on time to initiation of antiretroviral therapy: a before-after study. PLoS One. 2012;7(10):e46988 10.1371/journal.pone.0046988 23071690 PMC3465310

[33] Owiti P, Zachariah R, Bissell K, Kumar AM, Diero L, Carter EJ, et al Integrating tuberculosis and HIV services in rural Kenya: uptake and outcomes. Public Health Action. 2015;5(1):36–44. 10.5588/pha.14.0092 26400600 PMC4525370

[34] Onovo A, Kalaiwo A, Okechukwu E. One-Stop Shop: A Community-Based Antiretroviral Therapy (ART) Clinic Model to Improve Human Immunodeficiency Virus (HIV) Prevention and Treatment Cascade for Key Populations in Nigeria. Open Forum Infectious Diseases. 2016;3(suppl_1):483–.

[35] Patel P, Speight C, Maida A, Loustalot F, Giles D, Phiri S, et al Integrating HIV and hypertension management in low-resource settings: Lessons from Malawi. PLOS Medicine. 2018;15(3):e1002523 10.1371/journal.pmed.1002523 29513674 PMC5841643

[36] Craig P, Dieppe P, Macintyre S, Michie S, Nazareth I, Petticrew M, et al Developing and evaluating complex interventions: the new Medical Research Council guidance. BMJ. 2008;337:a1655 10.1136/bmj.a1655 18824488 PMC2769032

