# Supplementary material for: Capabilities, opportunities and motivations for integrating evidence-based strategy for hypertension control into HIV clinics in Southwest Nigeria
Source: PLoS One. 2019 Jun 6;14(6):e0217703. doi: 10.1371/journal.pone.0217703 (PMC6553742; doi:10.1371/journal.pone.0217703)
Supplement: S1 Text — (DOCX) [file pone.0217703.s001.docx]

ID Number: _______________

**Demographics**

1. Name of Health Facility:________________________________
2. Type of facility (check all that apply):


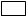
 Public
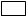
 Private
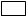
 Government
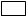
 Non-Government
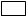
 Teaching

1. Age: _________
2. Sex: Male
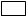
 Female
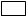

3. Highest level of education completed:


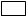
 Primary
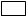
 Secondary
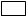
 Technical training
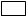
 Bachelors
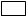
 Masters
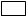
 Doctorate

1. Training:

| 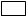Physician | 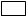Medical Officer | 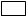Specialized Physician (specialty)___________ |
| --- | --- | --- |
| 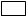Matron | 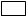Nursing/Midwifery | 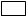Other (specify) ____________ |

1. How many years have you been providing HIV care? _________

***The following questions*** *assess the management and control of hypertension in your healthcare facility at the organizational, provider, and patient level.*

**Organizational**

1. How many HIV patients are seen in a month in your facility? ________________
2. Among the HIV patients, how many are hypertensive? ________________
3. Which of the following investigative approach is used for the assessment of hypertensive HIV patients?

|  | **Yes** | **No** | **Do not know** |
| --- | --- | --- | --- |
| Urine Analysis |  |  |  |
| Plasma creatinine or blood |  |  |  |
| Blood glucose |  |  |  |
| Serum electrolytes |  |  |  |
| Total cholesterol |  |  |  |
| Serum Lipoprotein |  |  |  |
| Electrocardiogram |  |  |  |
| Abdominal Ultrasound |  |  |  |
| Echocardiogram |  |  |  |

1. What anti-hypertensive drugs are available for the HIV patients at your facility?

Bendrofluazide Amlodipine
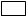
 Lisinopril
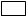
 Metoprolol
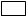
 Other (Please list) ________________

1. How often are anti-hypertensive drugs readily available at your facility?


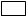
Very often
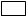
 Quite often
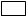
 Occasionally
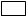
 Rarely
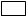
 Never

1. Are the anti-hypertensive drugs provided to patients free of charge?


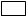
 Yes
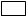
 No
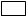
 Do not know

1. If no to question #13, how much do the patients pay for the medications (in Nigerian Naira)? ________________
2. How do most HIV patients at your facility cover the cost of hypertension treatment or consultation?


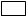
 Fully out of pocket
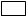
 Partial payment
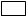
 Insurance
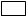
 Other (specify) ________________

**Healthcare Provider**

1. What is the first-line of anti-hypertensive drug choice for hypertensive treatment? ____________
2. What do you consider the blood pressure threshold for initiation of an anti-hypertensive treatment (please provide systolic and Diastolic numbers)? ____________
3. Who is responsible for ***identifying*** hypertensive HIV patients in your facility? (check all that apply)

| 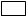Physician | 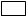Medical Officer | 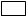Specialized Physician (specialty)___________ |
| --- | --- | --- |
| 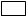Matron | 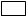Nursing/Midwifery | 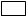Other (specify) ____________ |

1. Who is responsible for ***the treatment*** of hypertensive HIV patients in your facility? (check all that apply)

| 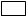Physician | 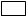Medical Officer | 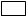Specialized Physician (specialty)___________ |
| --- | --- | --- |
| 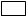Matron | 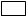Nursing/Midwifery | 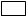Other (specify) ____________ |

1. Who is responsible for ***referring*** hypertensive HIV patients in your facility? (check all that apply)

| 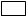Physician | 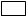Medical Officer | 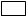Specialized Physician (specialty)___________ |
| --- | --- | --- |
| 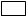Matron | 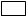Nursing/Midwifery | 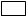Other (specify) ____________ |

1. Who is responsible for the ***follow-up care*** of hypertensive HIV patients in your facility? (check all that apply)

| 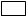Physician | 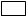Medical Officer | 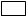Specialized Physician (specialty)___________ |
| --- | --- | --- |
| 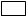Matron | 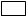Nursing/Midwifery | 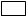Other (specify) ____________ |

1. How are patient’s anthropometric measurements taken in your facility? (check all that apply)


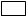
 Stethoscope
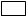
 Weighing scales
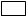
 Stadiometer
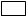
others (specify) ____________

1. How often are hypertensive patient’s blood pressures taken?


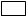
Very often
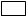
 Quite often
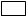
 Occasionally
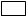
 Rarely
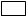
 Never

1. What type of Sphygmomanometers is used to check patient’s blood pressure?


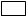
Mercury
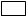
 Anaeroid
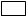
 Digital
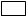
 Other (specify) ____________

**Patients**

1. What type of life-style related information does the facility provide to HIV patients for the management of hypertension? (check all that apply)

| 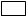 Heart-healthy diet | 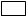 Reduce coffee/caffeine | 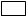Physical activity |
| --- | --- | --- |
| 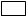 Low sodium intake | 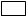 No tobacco products | 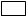Other (specify) _____________________ |

1. What are the responses of hypertensive HIV patients regarding the beneficial effects of the lifestyle-related information?

|  | **Beneficial** | **Not beneficial** | **Do not know** |
| --- | --- | --- | --- |
| Heart-healthy diet |  |  |  |
| No tobacco products |  |  |  |
| Physical activity |  |  |  |
| Low sodium intake |  |  |  |
| Reduce coffee/caffeinated drink intake |  |  |  |
| Other (specify) ____________ |  |  |  |

1. What are the sources of knowledge of the beneficial effects of living a healthy lifestyle among hypertensive HIV patients?


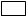
 Doctor
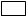
 Non-physician health-care provider
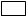
 Lay acquaintance
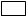
 Television or media

- Other (Specify)

**Organizational Context for Implementing Evidence-Based Practices for Hypertension Management and Control within HIV Clinics**

*This 14-item measure assesses the degree to which evidence-based practices for hypertension management and control are implemented within HIV clinics for patients living with HIV.*

Instructions: Please indicate the extent to which you agree with each statement.

| **0** | **1** | **2** |
| --- | --- | --- |
| Don’t Know | No | Yes |

**Focus on Evidence-Based Practice for Hypertension Treatment**

| 1. One of the main goals of this facility is to effectively use evidence-based practices for hypertension management and control in HIV patients | 0 | 1 | 2 |
| --- | --- | --- | --- |
| 1. Evidence-based practices for hypertension treatment are important to the providers in this facility | 0 | 1 | 2 |
| 1. Using evidence-based for hypertension treatment is a top priority for the providers in this facility | 0 | 1 | 2 |

**Educational Support for Evidence-based Practice for Hypertension Treatment**

| 1. The facility provides specific conferences, workshops or seminars on evidence-based practices for hypertension management and control | 0 | 1 | 2 |
| --- | --- | --- | --- |
| 1. The facility provides evidence-based practice for hypertension treatment in-service training | 0 | 1 | 2 |
| 1. Training materials, journals and other educational resources on evidence-based practices for hypertension management and control are provided | 0 | 1 | 2 |

**Recognition for Evidence-Based Practice for Hypertension Treatment**

| 1. Providers in this facility who utilize evidence-based practices for hypertension treatment are highly recommended | 0 | 1 | 2 |
| --- | --- | --- | --- |
| 1. The providers in this facility who utilize evidence-based practices for hypertension treatment are well respected among their peers | 0 | 1 | 2 |
| 1. Providers in this facility who utilize evidence-based practices for hypertension treatment are more qualified | 0 | 1 | 2 |

**Reward for Evidence-Based Practice for Hypertension Treatment**

| 1. Providers are financially incentivized to use evidence-based practices for hypertension treatment | 0 | 1 | 2 |
| --- | --- | --- | --- |
| 1. The more a provider use evidence-based practices, the more likely they are to get a commission | 0 | 1 | 2 |

**Selection for Openness**

| 1. This facility favors providers who are ***adaptable*** to the guidelines for hypertension treatment | 0 | 1 | 2 |
| --- | --- | --- | --- |
| 1. This facility favors providers who are ***flexible*** to the guidelines for hypertension treatment | 0 | 1 | 2 |
| 1. This facility favors providers who are open to new types of interventions for hypertension management and control … | 0 | 1 | 2 |

*What specific type of evidence-based practice training for hypertension management among HIV patients does your facility provide? How often? ________________________________________________________________*

*________________________________________________________________________________________________________________________________________________________________________________________________________________________________________________________________________________________________________________________________________________________________________________*
